# Neuralgia—A Case

**Published:** 1883-01

**Authors:** 


### Editorial.

### Neuralgia--a Case.

On the 28th of November Mrs. H., aged about 30, presented the first right inferior molar, for an operation. The tooth was not decayed, but was worn away by chemical abrasion, about two thirds from the original crown surface of the tooth to the gum; on the lingual side a little more than on the cervical. The exposed dentine was but slightly sensitive; the pulp was not exposed nor was the tooth sensitive over it; its covering was of secondary deposit. The request was for a capping upon this tooth that should arrest the wasting—for a capping only, and not for a full restoration of the crown. As no treatment was requisite the operation was taken up at once. The anchorages were made sufficiently outside the pulp chamber that it should not be encroached upon nor even affected; in this there was no difficulty, as there was ample material in which to secure the object. This being done the capping was built on in the usual manner, and need not here be described. It was finished and the patient dismissed.

Three days afterward the patient returned, stating that for the last twelve hours she had suffered intense pain, though none before,—a perfect agony, as she described it. She had not slept a moment during the night, had used hot water in the mouth, hot foot baths, and surface friction over the body and had taken 10 grains quinine, all this during the night, and that without any mitigation of the symptoms. She came in the morning for relief. Upon examination there was not the slightest inflammation apparent in the parts, no soreness of the tooth, nor of any of the neighboring teeth. The pain was sharp, intense and persistent, without break or intermission, seeming to have its seat in the jaw, between the second lower molar and the cuspid on the side in question, but reaching out in a sort of snatching way to the symphories on the one hand and to the ear and side of the head on the other. The treatment employed consisted in the application of highly stimulating preparations to the jaw and painting the gums with tincture Iodine, and 20 grains quinine during the next twentyfour hours, and hot foot-baths; and to report next morning. All of which was carried out to the letter, and especially the return in the morning, when the patient seemed greatly demoralized; had obtained no relief, and had slept none except an hour in the morning.

Upon examination the case was about as the day before. Applied counter-irritants and prescribed saline purgatives. Another twenty-four hours was passed with much the same condition, though more sleep was obtained during the night, rather because of exhaustion than mitigation of the pain. On the third day in the afternoon the patient called again, with no special improvement in the symptoms. Removal of the capping of gold was suggested, as was also drilling into the pulp chamber and destroying the pulp; to both of these, however, the patient objected.

The second bicuspid was gone and on the posterior proximate surface of the first bicuspid there was a large amalgam filling. This tooth had never given any pain or inconvenience. The tooth was somewhat decayed about the amalgam filling. It had been suggested that this tooth should be refilled. At the third visit the patient expressed the suspicion that the amalgam filling might have something to do with this trouble, and she requested that the filling be at once removed which was done. The tooth in the cavity was not sensitive in any respect.

Within one minute after the removal of the amalgan the patient exclaimed, there! there is not a particle of pain; it has wholly disappeared. She remained for perhaps twenty minutes awaiting further results; not a twinge of pain occurring during this time. She returned home and remained away two days, then returned and reported that no pain or even soreness had been experienced. The cavity from which the amalgam had been removed was excavated and filled with oxyphosphate of zinc, and during the subsequent six weeks no return of pain or any unpleasant symptoms occurred. Now what was the difficulty? Was it galvanic action? If so, what was the conductor? Neither the gold capping nor the amalgam filling was in contact with the gum, each being nearly a line from it. The secretions of the mouth were in a healthy condition. If there was a galvanic current, did the mucous membrane or the moisture, or both, conduct, or was the nerve connection between the two teeth the conductor? The latter seems the more probable, and is the only plausible theory upon which to account for the severe, prolonged and continuous pain. But upon this theory the current must have passed through a considerable thickness of dentine in each tooth. How about that? Will somebody tell the readers of the REGISTER. Would any other treatment have been available than the removal of one or the other of the metals, or destroying one of the pulps?

I have seen many cases like this in some respects, but never one similar in all the features. I now yield to the man who knows more about such things.

